# Lymphoma presenting as gynaecomastia

**DOI:** 10.2349/biij.7.2.e10

**Published:** 2011-04-01

**Authors:** S Mahmood, Z Sabih, D Sabih

**Affiliations:** Multan Institute of Nuclear Medicine and Radiotherapy, Multan, Pakistan

**Keywords:** Breast imaging, lymphoma, gynaecomastia

## Abstract

Breast lymphoma is an uncommon neoplasm affecting the breast and is extremely rare in males. While gynaecomastia is common and in most cases benign, it can sometimes result from significant pathology and the physician should keep in mind the possible diseases that can lead to gynaecomastia. This paper reports a case of lymphoma presenting as unilateral gynaecomastia. The paper discusses the differential diagnosis and emphasises the points that should raise the suspicion of pathology.

Mammography, high resolution ultrasound and biopsy findings are discussed and literature survey is presented.

## INTRODUCTION

Gynaecomastia refers to enlargement of the breasts in males. It is very common and one author has reported a prevalence of 57% in the studied normal population [1]. It is usually benign and is thought to be due to an altered oestrogen-androgen balance or from increased breast sensitivity to normal circulating oestrogen levels [2]. This can be unilateral or bilateral, and some cases can be pathologic. While most cases are physiologic, pathologic gynaecomastia will require further evaluation.

Differentiation between benign and malignant masses is critical because it will avoid unnecessary procedures. Breast lymphoma, either as a manifestation of primary extranodal disease or as secondary involvement, is rare, and relatively small groups of patients are reported in the literature. The reported incidence of breast lymphoma ranges from 0.04% to 0.5% of all breast malignancies [3]. Only 19 cases were reported till 2004 [4].

Because the radiographic features of breast lymphoma are nonspecific, the diagnosis of primary breast lymphoma cannot be made on the basis of mammographic findings alone. Diagnostic work-up includes bilateral mammography, high resolution ultrasound, and histopathology.

## CASE REPORT

**Figure 1:**
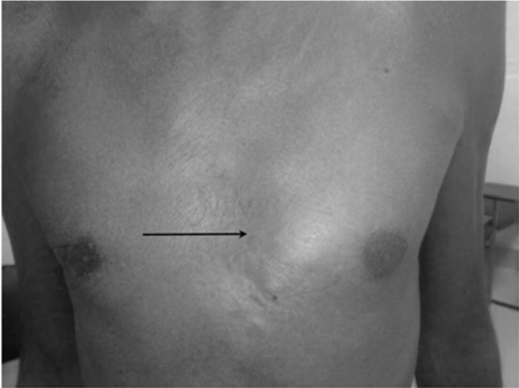
Photograph of the patient, note the left sided Gynaecomastia (arrow).

A 50-year-old male patient presented with unilateral breast enlargement for 4 months (Figure 1). On examination, there was a 2 × 1 cm lump in the left breast just behind the nipple. The lump was mobile, non-tender, and not fixed to the skin or underlying muscles. Renal and liver function tests were normal. Scrotal ultrasound was unremarkable.

**Figure 2:**
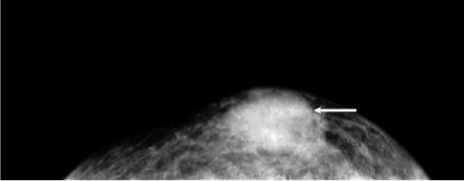
Mammogram, craniocaudal view showing the left retroareolar, high density, radioopaque opacity (arrow). There are no skin changes or nipple retraction.

**Figure 3:**
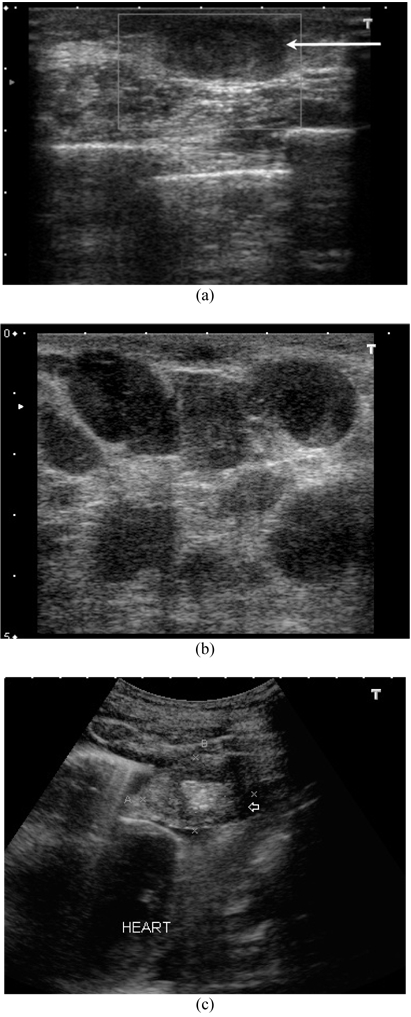
a) Ultrasound, transverse section through the palpable lump. Note the hypoechoic nodule (arrow) with micro lobulation. The Doppler colour box shows no blood flow signals; b) Ultrasound of the left axilla. Note cluster of enlarged nodes; these are round and have lost the central echogenic hilum suggesting malignant infiltration; c) Ultrasound through a left intercostal space, showing a parietal mass in the left pleural cavity (arrow) that touched the heart on real-time scanning.

Mammography was done and craniocaudal and mediolateral oblique views were acquired. On mammography (Figure 2) there was a solitary, high density, irregular, uncalcified opacity in the retroareolar region of left breast. No skin or nipple changes were seen. On ultrasound (Figure 3), there was a hypoechoic mass with microlobulations measuring 2.7 cm × 1.5 cm in the left retroareolar area. Doppler did not show any significant vascularity. There were multiple axillary lymph nodes that showed loss of the normal reniform appearance. There were several nodules within and deep to the left pectoralis major muscle. The left supracalvicular nodes were enlarged. A small nodule was seen within the right pectoralis major, adjacent to the sternum. Another mass was seen extending into the left pleural space and abutting onto the heart. There was a large left pleural effusion.

**Figure 4:**
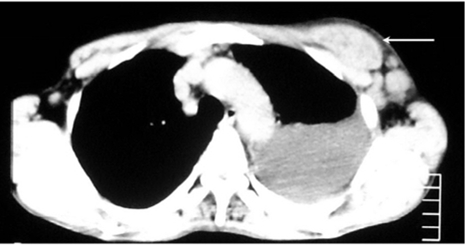
CT scan through the thorax, showing the chest wall mass (arrow).

**Figure 5:**
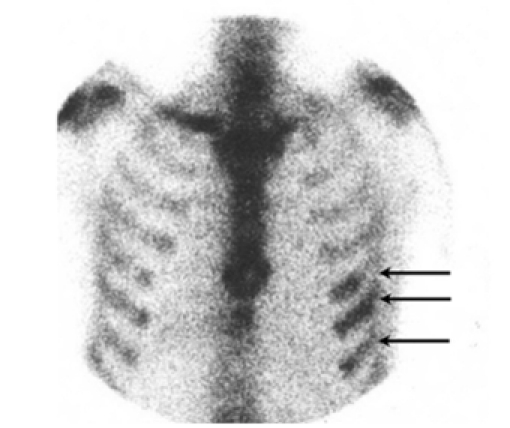
Bone scan, anterior thorax. This is largely unremarkable except for increased uptake in the anterior ends of the left sided 6th – 8th ribs (arrows).

Computed tomography (Figure 4) showed a soft tissue mass within the left chest wall and left pleural effusion. Bone scan (Figure 5) showed diffuse increased uptake in the anterior ends of the left 6th -8th ribs. Left axillary lymph node biopsy was done and showed large B-cell lymphoma. On immunohistochemistry, the lymphoid cells were diffusely positive for CD20. Fine needle aspiration cytology of breast lump showed atypical round cells, consistent with non-Hodgkin’s lymphoma. Bone marrow aspiration showed good cellularity with no infiltration.

## DISCUSSION

Gynaecomastia is the development of abnormally large mammary glands in males, resulting in breast enlargement. Gynaecomastia is not usually clinically significant, but in some cases can be an indicator of serious underlying conditions. It has tri-modal peaks at infancy, adolescence and old age. Although the term refers specifically to benign overgrowth of breast parenchyma in males, any breast enlargement in males is called gynaecomastia.

Growing glandular tissue, typically from some form of hormonal stimulation, is often tender or painful, and accompanied by social and psychological difficulties for the sufferer [5]. Gynaecomastia results from an altered oestrogen-androgen balance, in favor of oestrogen, or from increased breast sensitivity to a normal circulating oestrogen level [2]. The imbalance is between the stimulatory effect of oestrogen and the inhibitory effect of androgen. Oestrogens induce ductal epithelial hyperplasia, ductal elongation and branching, proliferation of the periductal fibroblasts, and an increase in vascularity. The histologic picture is similar in male and female breast tissue after exposure to oestrogen [6].

In a case of gynaecomastia, further workup is needed if the breast size is greater than 5cm (macromastia), the breast or breast lump is tender, has appeared recently, is growing or is hard with enlarged nodes or positive lymph node findings.

Unilateral Gynaecomastia is physiological in most cases but merits more careful evaluation for significant disease. Common causes of unilateral Gynaecomastia are given in Table 1. The majority of axillary lymphadenopathy is due to benign conditions such as reactive hyperplasia, infection, or granulomatous disease. The most common causes of malignant lymph nodes include lymphoma and metastatic disease from breast cancer, lung cancer, melanoma, and squamous cell cancer [7].

**Table 1:** Causes of unilateral gynecomastia

**Condition**	**Clinical features**	**Imaging features.**
Gynaecomastia	Palpable lump with nipple tenderness	Nodular subareolar tissue density on mammography Hypoechoic subareolar fan-shaped mass surrounded by normal fatty tissue
Abscess	Lump or induration with signs of inflammation	High density, Irregular opacity on mammography Irregular, fluid consistency lesion on ultrasound
Intra-ductal carcinoma	Lump with skin or nipple inversion, Peaude orange skin appearance	High density, irregular, spiculated mass with calcifications Hypoechoic, irregular, solid mass on ultrasonography
Metastatic disease to breast (melanoma, sarcoma, CA lung, CA stomach)	Multiple bilateral lumps	Bilateral irregular, spiculated masses
Breast lymphoma	Solitary painless lump or diffuse rapid breast enlargement	Oval, high density masses on mammography Hypoechoic circumscribed or microlobulated mass on sonography

Primary breast malignancy accounts for less than 1% of the total cases. Other conditions can arise from the skin, subcutaneous fat, blood vessels, lymphatics, and nerves [8]. Male breast cancer accounts for 0.7% of total breast cancers [9]. Approximately 85% of primary male breast cancers are invasive ductal carcinoma [10]. Most men referred for breast imaging have palpable lumps, breast enlargement, or tenderness.

Workup involves blood chemistry for renal or liver diseases, and endocrine assays with testosterone, luteinising hormone, estradiol and dehydroepiandrosterone sulphate. Initial imaging tests like ultrasound of the testes, mammogram and breast ultrasound are carried out.

Most breast lymphomas present as oval-shaped, high-density, poorly circumscribed, uncalcified irregular or spiculated masses on mammography and as hypoechoic masses with microlobulated or irregular margins on sonography [11]. The sonographic features of malignant lymph nodes include an irregularly thickened cortex and distorted or absent fatty hila [10].

Primary breast lymphoma (PBL) is an uncommon neoplasm of the breast with an incidence of less than 0.6% of primary breast malignancies[12]. It is thought to develop from the lymphatics or intramammary lymph nodes. Most affected women present with this condition in the sixth or seventh decade of life, but patients as young as 12 years have been reported [13]. Incidence of breast lymphoma in men is extremely low. Fewer than 20 cases were reported till 2004 [4]. Forty-four percent of cases of breast lymphoma are primary, although 22% are manifestations of disseminated disease and 29% represent recurrence of preexisting lymphoma [14].

In the case described in this paper, the most information was obtained from mammography and ultrasound, with CT contributing no new information. Bone scan showed significant rib uptake but this could be secondary to increased vascularity because of the multiple parietal masses.

The diagnostic criteria for primary breast lymphoma, established by Wiseman and Lao in 1972, require a technically adequate specimen, no evidence of spread of disease, and no previous diagnosis of extra-mammary lymphoma [15]. The patient in this case had positive axillary nodes and there were multiple nodules in the left chest wall, an intrathoracic mass as well as left pleural effusion, so it is believed that this was an unusual manifestation of disseminated disease.
